# Structure-Guided Design Affirms Inhibitors of Hepatitis C Virus p7 as a Viable Class of Antivirals Targeting Virion Release

**DOI:** 10.1002/hep.26685

**Published:** 2013-12-24

**Authors:** Toshana L Foster, Gary S Thompson, Arnout P Kalverda, Jayakanth Kankanala, Matthew Bentham, Laura F Wetherill, Joseph Thompson, Amy M Barker, Dean Clarke, Marko Noerenberg, Arwen R Pearson, David J Rowlands, Steven W Homans, Mark Harris, Richard Foster, Stephen Griffin

**Affiliations:** 1 Leeds Institute of Cancer & Pathology & CRUK Clinical Centre, Faculty of Medicine and Health, St James’ University Hospital, University of LeedsLeeds, West Yorkshire, UK; 2 School of Molecular & Cellular Biology & Astbury Centre for Structural Molecular Biology, Faculty of Biological Sciences, University of LeedsLeeds, West Yorkshire, UK; 3 School of Chemistry, Faculty of Mathematics and Physical Sciences, University of LeedsLeeds, West Yorkshire, UK; 4 School of Medicine, King’s College LondonLondon, UK; 5 Department of Oncology, University of OxfordOxford, UK; 6 Executive Office, Newcastle UniversityKing’s Gate, Newcastle upon Tyne, UK

## Abstract

Current interferon-based therapy for hepatitis C virus (HCV) infection is inadequate, prompting a shift toward combinations of direct-acting antivirals (DAA) with the first protease-targeted drugs licensed in 2012. Many compounds are in the pipeline yet primarily target only three viral proteins, namely, NS3/4A protease, NS5B polymerase, and NS5A. With concerns growing over resistance, broadening the repertoire for DAA targets is a major priority. Here we describe the complete structure of the HCV p7 protein as a monomeric hairpin, solved using a novel combination of chemical shift and nuclear Overhauser effect (NOE)-based methods. This represents atomic resolution information for a full-length virus-coded ion channel, or “viroporin,” whose essential functions represent a clinically proven class of antiviral target exploited previously for influenza A virus therapy. Specific drug-protein interactions validate an allosteric site on the channel periphery and its relevance is demonstrated by the selection of novel, structurally diverse inhibitory small molecules with nanomolar potency in culture. Hit compounds represent a 10,000-fold improvement over prototypes, suppress rimantadine resistance polymorphisms at submicromolar concentrations, and show activity against other HCV genotypes. *Conclusion*: This proof-of-principle that structure-guided design can lead to drug-like molecules affirms p7 as a much-needed new target in the burgeoning era of HCV DAA. (Hepatology 2014;59:408–422)

Many global viral pathogens encode essential ion channel proteins, so-called “viroporins,” representing an important opportunity for antiviral development. Clinical precedent was set by adamantane inhibitors of influenza A virus (IAV) M2 in the 1960s, yet widespread resistance limits their current use. However, viroporins retain potential as antiviral targets amenable to modern-day drug development.

Hepatitis C virus (HCV) infects ∼170 million individuals, causing severe liver disease and hepatocellular carcinoma (HCC). HCV encodes a cation-selective viroporin, p7,^1^–^3^ which is essential *in vivo*^4^ and required for efficient secretion of virions.^5^,^6^ p7 has been described to perform at least two distinct functions in culture: an early role interacting with NS2,^7^–^11^ as well as a late-acting function as an oligomeric proton channel that dissipates pH gradients in secretory vesicles, potentially protecting acid-labile intracellular virions during egress.^12^ Several prototype p7 inhibitors block channel activity and release of infectivity in a strain-dependent fashion.^13^–^16^ However, prototype potency is low and clinical trial data have been unconvincing.^17^,^18^ Recently, we identified specific resistance polymorphisms that indicated the likely modes of action for two p7 inhibitor prototypes: adamantanes targeted a membrane-exposed allosteric site on the channel periphery to stabilize closed channel complexes, whereas nonylated imino-sugars prevented channel oligomerization.^15^

A lack of atomic structural information has hindered the development of bespoke p7 inhibitors. p7 comprises two *trans*-membrane (TM) helices and forms hexameric and/or heptameric channel complexes,^1^,^19^,^20^ with activity influenced by membrane composition.^21^ Elegant electron microscopy reconstructions (16 Å resolution) revealed flower-like hexameric complexes formed by genotype 2a p7 in detergent micelles,^20^ although short detergent chain length may broaden this structure.^22^ A conserved His17 (primarily genotypes 1, 3, and 4) has been shown biochemically to line the channel lumen,^23^ comprising the N-terminal TM region. p7 structural investigations have been undertaken using both solution-nuclear magnetic resonance (sNMR) and solid-state NMR (ssNMR). sNMR in 50% tri-fluoro-ethanol (TFE) yielded secondary structure elements, but no information on 3D fold or long-range interactions, limiting outputs to a molecular dynamics (MD) model.^24^ ssNMR studies support models where p7 TM domains split into four separate helical regions, tilted relative to the membrane normal,^25^,^26^ yet a full structure remains elusive.

Here we describe the complete sNMR structure of monomeric FLAG-tagged p7 (FLAG-p7) in a structurally relevant hairpin conformation. Supported by specific protein-drug interactions, this structure serves as an accurate template for the selection of novel, highly potent virion release inhibitors. Modified derivatives based on initial hits suggest that subsequently derived lead molecules could lead to an HCV-targeted drug, creating a valuable addition to future drug combinations necessary to combat the emergence of resistant HCV.

## Materials and Methods

### Isotopically Labeled FLAG-p7

Expression and purification (reverse-phase high-performance liquid chromatography [rp-HPLC]) of FLAG-p7 has been described.^13^,^15^,^19^,^27^,^28^ *Escherichia coli* BL21(DE3) transformed with pGEX-FLAG-p7 (genotype (GT)1b J4 isolate wild-type, L20F) were grown at 30°C until an OD_600_ of 0.8 in M9 minimal media, with 15N ammonium chloride (1 g/L), 13C glucose (2 g/L), 0.04% FeCl(III), and BME vitamins (Sigma-Aldrich). Expression was induced overnight with shaking at 30°C using 1 mM IPTG.

### NMR Spectroscopy and Structure Calculation

A detailed description of NMR experiments and structure calculation protocols is provided in the Supporting Experimental Procedures section. NMR analysis of [^13^C, ^15^N]-FLAG-p7 (0.3-0.6 mM) in 100% MeOH was conducted using Varian Inova 500, 600, or 750 (cold-probe) MHz spectrometers at 25°C. Structure calculation employed a novel protocol where a chemical shift derived structure was first calculated using cs-memrosetta,^29^ providing secondary structure characteristics. Semirigidified secondary structural elements were then refined against observed nuclear Overhauser effects (NOEs) using Aria 2.3.^30^ Table 1 shows NMR and refinement statistics.

**Table 1:** NMR and Refinement Statistics for Protein Structures

	Protein
NMR distance and dihedral constraints	
Distance constraints	
Total NOE	512 restraints from 304 peaks
Intra-residue	214
Inter-residue	298
Sequential (|*i* – *j*| = 1)	106
Medium-range (|*i* – *j*| ≤ 4)	161
Long-range (|*i* – *j*| > 4)	61
Total dihedral angle restraints	104
ϕ	52
ψ	52
Structure statistics	
Violations (mean and s.d.)	
Distance constraints (Å)	0.0078 ±0.0003
Dihedral angle constraints (°)	1.3 ± 0.7
Max. dihedral angle violation (°)	2.2 ± 0.6
Max. distance constraint violation (Å)	0.0 0.0
Deviations from idealized geometry	
Bond lengths (Å)	0.00323 ± 0.00009
Bond angles (°)	0.560 ± 0.006
Impropers (°)	0.462± 0.016
Average pairwise r.m.s. deviation* (Å)	
Heavy	0.876
Backbone	0.306

* Pairwise r.m.s. deviations were calculated over residues −16 to 57 from the best 20 refined structures sorted by energy.

### Structure-Guided p7 Channel Models

Models were generated manually within Maestro (Schrodinger). Seven monomers were colocated around a centroid based on a requirement for common distance and angle between common atoms on individual monomers. The monomer stack was then tilted to allow for pore constriction at the loop region, and tilt towards adjacent monomers required for packing. The model was then symmetrized using the measuring tool and the angle-adjust options with respect to the centroid. Models were energy minimized using the MMFF tool in Macromodel in an octanol environment. Iterative refinement generated a model with the lowest global energy. Models for other genotypes were derived from the J4 model with subsequent energy minimization. Lumenal diameters were calculated using the “Hollow”^31^ and Pymol (DeLano Scientific).

### *In Silico* Compound Selection

A commercially available compound library (250K+ compounds) was screened against one of the seven allosteric binding sites present on the structure-guided channel model, defined by Leu20, using eHiTS (SymBioSys). The highest-ranking 2,000 were then redocked using eHiTS (high accuracy). Compounds were selected by raw eHiTS scores, requirement for drug-likeness, and lack of reactive functionality. The top 30 compounds were redocked in Glide (Schrodinger) and these coordinates used for the modeling studies described herein.

### *In Vitro* Assays for p7 Channel Activity

Liposome carboxyfluorescein release assays were conducted as described previously.^13^,^15^,^28^

### HCV Culture

Huh7 cells were maintained, transfected, and treated with inhibitors for 72 hours as described.^13^,^15^ Experiments employed JFH-1 (genotype 2a) subgenomic firefly luciferase replicon, full-length JFH-1, or chimeras encoding C-E1-E2-p7-NS2 proteins from other genotypes: 1b (J4), H77 (1a) J6 (2a), S52 (3a), and ED43 (4a). J4/JFH-1 Leu20Phe was generated by polymerase chain reaction (PCR) mutagenesis (details available upon request). Commercially available MTT (3-(4,5-dimethylthiazol-2-yl)−2,5-diphenyltetrazolium bromide) toxicity assays were carried out according to the manufacturer’s instructions (Roche).

### Protein Analysis

Western blots of Huh7 lysates and immunofluorescence analysis at 72 hours posttransfection used rabbit anti-core (308), mouse anti-E2 (AP33), rabbit anti-NS2, sheep anti-NS5A, and mouse anti-glyceraldehyde 3-phosphate dehydrogenase (6CS, Invitrogen), with appropriate horseradish peroxidase-conjugated (Sigma) or Alexa-Fluor conjugated (Invitrogen) secondary antibodies. Protocols as described.^13^,^32^

## Results

### Solution Structure of Monomeric FLAG-p7

sNMR was conducted in methanol (MeOH), reconstitution in which preserves FLAG-p7 in a functional, drug-sensitive state upon introduction to membranes (Supporting Fig. S1a).^13^,^28^ Importantly, biochemical and biophysical analysis confirmed FLAG-p7 to be both monomeric and in a helical fold when reconstituted in MeOH (intrinsic tryptophan fluorescence quenching, sedimentation velocity analytical ultracentrifugation, Fig. S1b-d). MeOH was preferable to 50% TFE, reconstitution in which reduced both p7 functionality and compound sensitivity in subsequent dye release assays (data not shown), as well as to 1,2-diheptanoyl-*sn*-glycero-3-phosphocholine (DHPC) which induced FLAG-p7 oligomerization in our hands (Fig. S1b,d). Critically, labeled FLAG-p7 was shown to form a hairpin by paramagnetic relaxation enhancement (PRE) (Fig. S1e). However, data were excluded from structure calculations due to chemical shift changes induced by both mutagenesis (Cys0 added at p7 amino-terminus, with C27S) and labeling (MTSL reagent added to either Cys0/27) (data not shown).

FLAG-p7 yielded well-resolved, fully assignable, dispersed ^1^H-^15^N correlation spectra consistent with a predominant helical fold (Fig. S2d,e). While *de novo* structure calculations yielded hairpin structures, orientation of the C-terminus relative to the N-terminal helix could not be unambiguously defined (Fig. S2a). Therefore, a chemical shift-based model of FLAG-p7 secondary structure elements was retained as semirigid helical domains refined against the 512 observed NOE constraints during the Aria structure calculation protocol, allowing all other degrees of freedom to evolve. The final series of 20 calculated structures were highly convergent (Fig. S2b) and showed a backbone RMSD of 0.306, with 61 long-range NOE constraints defining the hairpin (Fig. S2c). Residues in the FLAG tag did not interact with the biological unit (Fig. S2c).

**Figure 1:**
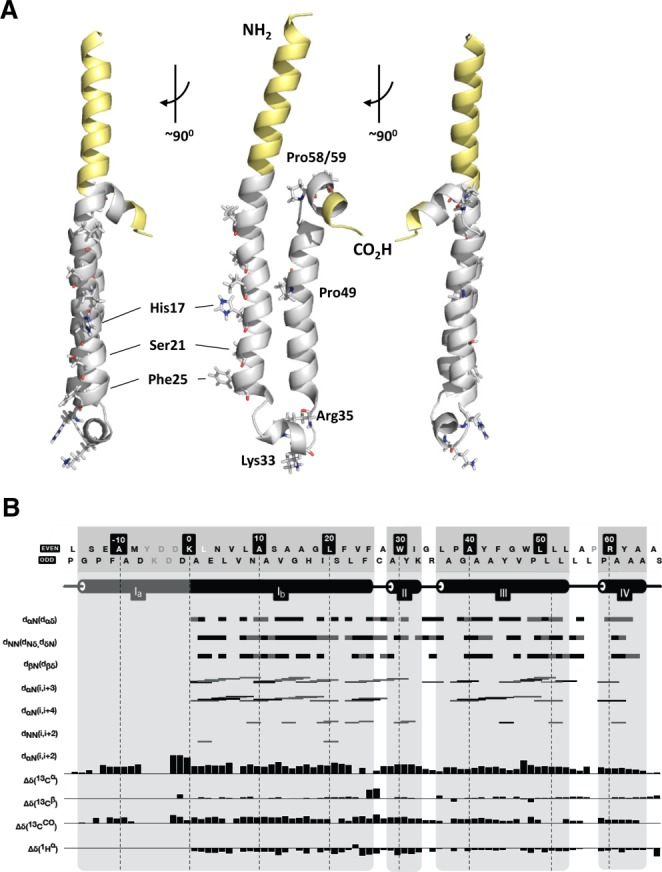
Solution NMR structure of FLAG-p7. The structure of isotopically labeled FLAG-p7 (GT1b) was calculated using a combined chemical shift/NOE methodology (see Materials and Methods). (A) Ribbon diagram of lowest energy structure for monomeric FLAG-p7. Yellow, FLAG-tag and polylinker sequence; gray, biological unit. Sidechains for key residues are shown, including His17, Ser21, and Phe25 predicted to form part of the lumenal surface in the channel complex,^15^,^22^,^23^ the conserved basic residues Lys33 and Arg35, Pro49 which forms a kink in the C-terminal helix. (B) Secondary structure characteristics of calculated solution NMR structure. Black bars show unambiguous restraints, gray bars show restraints that are detectable but with more than one possible assignment.

The lowest energy calculated p7 structure (Fig. 1A) is entered into the Protein Data Bank (ID: 3ZD0), and NMR data deposited (BMRB code: 18863). The first leg of the hairpin comprises a p7 α-helix (Ib) along with the α-helical flag tag (Ia), separated from the two C-terminal α-helices (III, IV) by a turn-helix-(II)-turn motif (Fig. 1B). The N-terminal helix (amino acids [aa] 1-25, p7 sequence) orients such that the His17 and nucleophilic Ser21 residues are aligned, consistent with pore-lining orientation.^15^,^22^–^24^,^27^ This “lumenal” face of the helix also contains a run of N-terminal small/hydrophobic residues and Phe25 at the cytosolic end, which may act as a molecular gate based on hyperactive Phe25Ala mutants.^15^ The interhelical loop extends from aa 26-37, and includes a short helical stretch from Ala29 to Lys33. The p7 C-terminus comprises a longer helix (aa 37-57) kinked at Pro49, and another short, highly flexible helix following a di-Pro motif (Pro58/59). The *trans*-membrane region was predicted by “Octopus” (see Supporting Experimental Procedures, Ref.^8^) to comprise Asn9-Ala29 (N-terminus) and Ala37-Leu57 (C-terminus). The overall structure more closely resembled predictions from ssNMR with regard to helical length, orientation, and the length of the interhelical loop compared with sNMR models, suggesting that MeOH induces a fold analogous to that observed in lipid-like detergents.^25^,^26^

### Drug-Protein Interactions Validate the Monomeric FLAG-p7 Hairpin Fold

Our previous *in silico* modeling predicted that the rimantadine binding site primarily comprises residues from both helices of a single folded p7 hairpin, centered on the Leu20 residue.^15^ Thus, despite clear differences between *in silico* models and our NMR structure, we hypothesized that drug interactions with both helices in solution would indicate that an analogous peripheral drug binding site existed, as well as corroborating the evidence supporting the FLAG-p7 hairpin fold (Fig. S1e).

**Figure 2:**
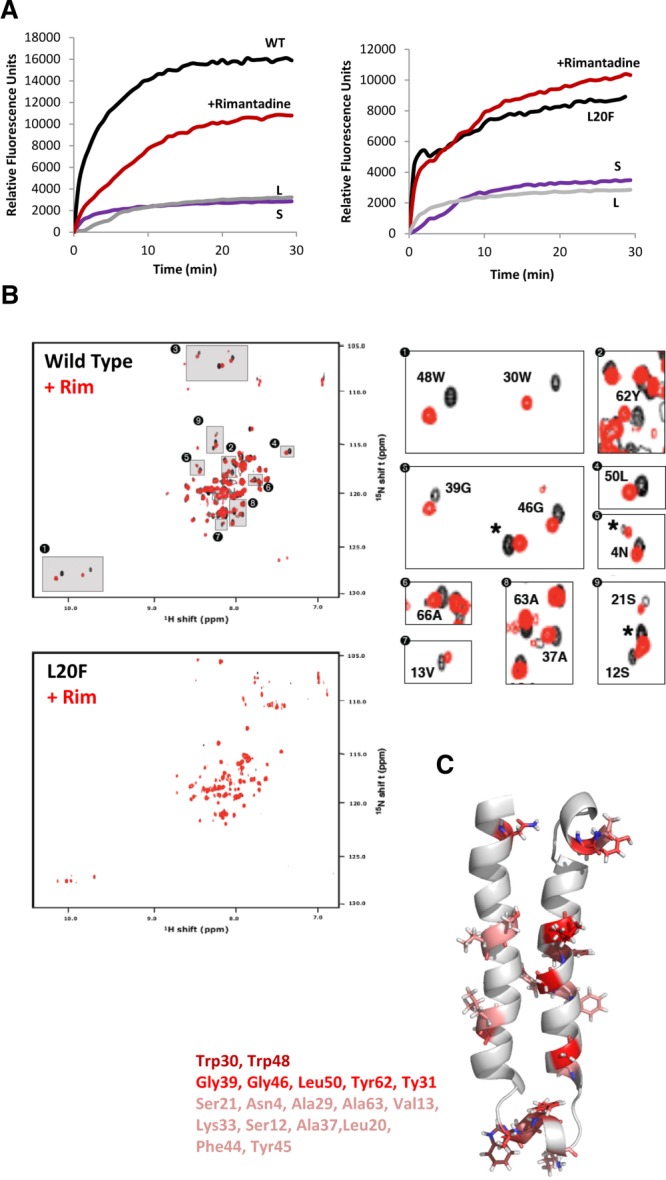
Validation of p7 NMR structure in methanol using specific drug interactions. (A) Isotopically labeled wild-type or Leu20Phe FLAG-p7 was tested for rimantadine sensitivity in dye release assays. Black lines, protein only; red line, protein plus 40 µM rimantadine; L, liposomes only; S, solvent control. (B) ^1^H/^15^N HSQC spectra of wild-type and Leu20Phe p7 in the presence (red) or absence (black) of a 2 molar excess of rimantadine. Specific minimal chemical shift changes in the presence/absence of drug were detectable in wild-type, but not Leu20Phe resistant protein (see Supporting Fig. 2c for chemical shift change in parts per million (Δppm) values). (C) Positions of residues on monomeric structure seen to experience chemical shift changes in the presence of rimantadine, including Leu20. Graded color represents magnitude of Δppm; pink (0.14-0.25), red (0.25-0.5), dark red (0.5+).

We used a variant of NMR conservative chemical shift mapping to investigate drug interactions, calculating a shift metric (Δppm) from minimal isotope-weighted chemical shift changes that map peaks from an assigned (apo/unbound) state spectrum onto those of the unassigned (holo/drug-bound) state.^33^ Chemical shift changes could occur through direct ligand interactions, or by way of structural/dynamic changes upon binding. For FLAG-p7 interacting with rimantadine, Δppm ≥0.14 were deemed significant, with shift changes of up to ∼0.8 ppm observed for some residues (Fig. S3). To gauge specificity, interactions were repeated for Leu20Phe FLAG-p7,^15^ which conferred resistance to isotopically labeled protein in dye release assays (Fig. 2A). Accordingly, rimantadine induced significant Δppm in the region neighboring Leu20 for wild-type, but not Leu20Phe FLAG-p7, consistent with this polymorphism directly preventing adamantane binding (Fig. 2B). Accordingly, Leu20 was among a number of residues from both helices in the central portion of the hairpin experiencing significant shifts (Fig. 2B,C), with Trp48, Leu50, and Gly46 also showing particularly clear perturbations. Thus, while precise composition of the Leu20-defined rimantadine binding site differed from that predicted by *in silico* models,^15^ it comprised many of the same key residues, was located at a similar position, and would likely face the channel periphery in the context of an oligomer. Importantly, the site comprised several residues with a high degree of conservation across genotypes, such as Gly46, Trp48, and Leu50. Interestingly, several additional shifts were apparent in the loop region and near the N/C termini of the protein, which were absent for Leu20Phe (Fig. 2B,C). It was not possible to determine whether these represent additional interaction sites, or result from changes in the overall structure of the molecule following rimantadine binding. However, shifts were dependent on initial rimantadine binding in the vicinity of Leu20, supporting this as the primary adamantane interaction surface. Furthermore, the composite nature of this binding site supports that FLAG-p7 adopts a hairpin fold in MeOH.

### Generation of Structure-Guided p7 Channel Models as *In Silico* Screening Templates

**Figure 3:**
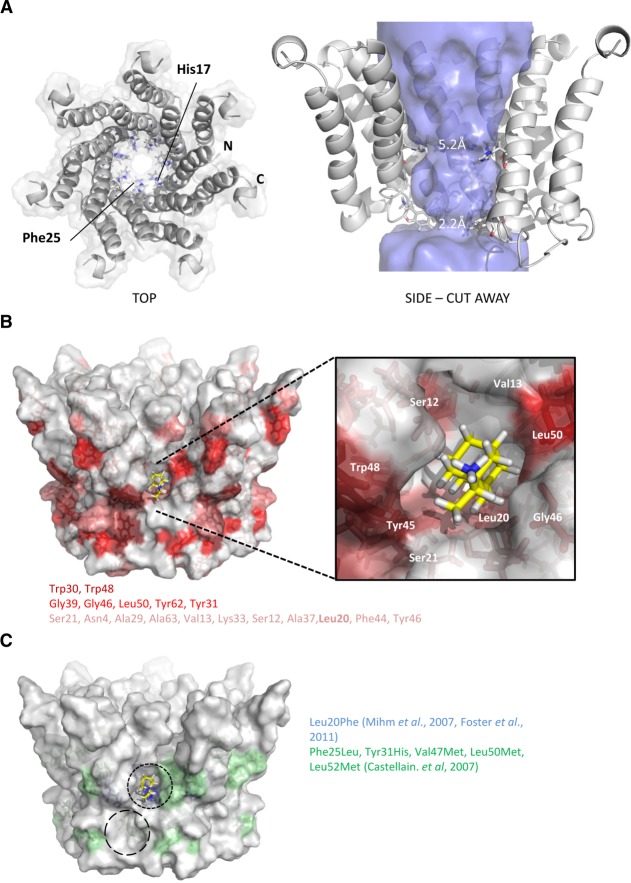
Construction of heptameric channel models based on monomeric p7 NMR structure. Energy minimized channel models were constructed in Maestro (see Materials and Methods) by symmetrical arrangement of individual biological unit protomers with multiple rounds of energy minimization. Stable structures resulted from a tilted orientation of individual protomers relative to the predicted membrane normal. The N-terminal helices formed the channel lumen and the C-terminal helices stabilized the channel complex, consistent with previous models and predictions. (A) Left: top-down surface view showing individual protomers as ribbons. His17 and Phe25 are highlighted projecting into the lumen. Right: side view cutaway showing orientation of individual protomers, His17 and Phe25 highlighted and lumenal diameter indicated by the “Hollow” program.^31^ (B) Residue positions in context of heptameric channel model seen to interact in NMR experiments (color grading as in Fig. 2C). (C) Amino acid changes in patients nonresponsive to amantadine in two separate studies as indicated. Predicted rimantadine docking is shown for one of the seven primary peripheral binding pockets (black dashed line) along with a potential secondary binding site (longer dashed circle).

The monomeric structure was used to generate an energy-minimized heptameric model of the assembled p7 channel complex as a template for *in silico* compound design. Energetically stable complexes resulted from p7 protomers being tilted with respect to the membrane normal, consistent with ssNMR^26^ (Fig. 3A). Models retained the predicted lumenal orientation of His17 and Phe25 (Fig. 3A) which caused lumenal constrictions, and were more energetically stable compared to previous *de novo* models^15^,^27^ (NMR: −54,472 kcal/mol; *de novo*: −17,806 kcal/mol). Critically, the allosteric adamantane binding site was apparent on the channel periphery (Fig. 3B, black dotted circle) and contained many rimantadine-interacting residues (Figs. 2C, 3B, darker red indicating stronger changes). Sites included Leu20 at the base of the pocket (Fig. 3B,C), which, combined with its observed shift change in the presence of drug (Fig. 2B,C) provides clear rationale underpinning Leu20Phe rimantadine resistance in genotype 1b.^15^,^34^ Interestingly, loop-proximal residues observed to shift in the presence of rimantadine (Fig. 2C) centered on another peripheral cavity (Fig. 3C, long-dashed circle). These overlapped with nonsynonymous polymorphisms in other genotype 1b patients unresponsive to amantadine^35^ (Fig. 3C). It is possible that both peripheral cavities interact with adamantanes, although the Leu20Phe phenotype supports a primary role for the original site, which accommodates rimantadine molecules more favorably than the second site (Glide, >2 log_10_ difference). Thus, convergence of the structure-guided model with observed/predicted drug interactions supports the relevance of the FLAG-p7 monomer structure.

### Structure-Guided Models Select p7 Inhibitors With Genuine Potency

**Figure 4:**
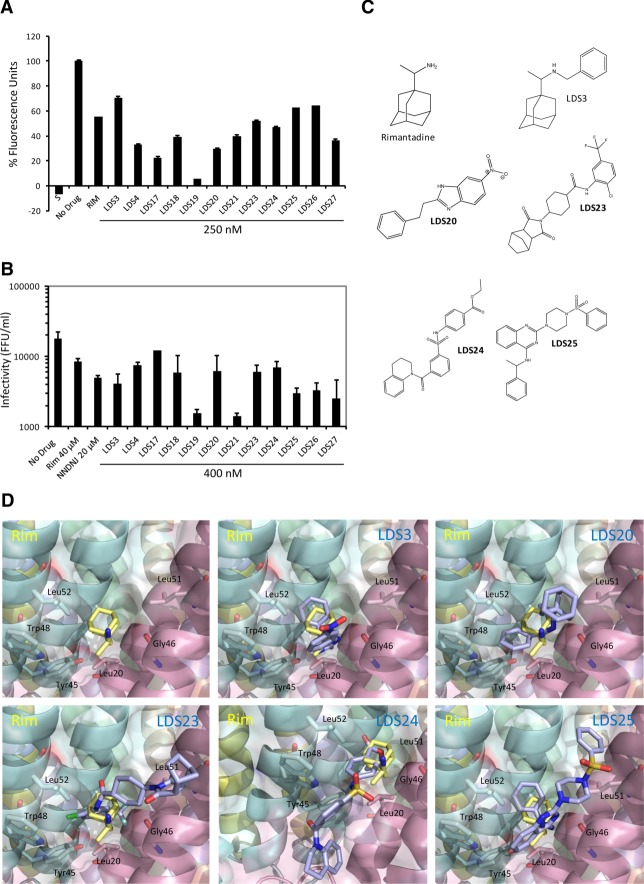
Selection of novel, structurally diverse p7 inhibitors. *In silico* screening using p7 channel models as a template derived a range of Lipinski-compliant potential inhibitors of the p7 channel complex and the top 30 were selected for testing as proof-of-principle. (A) Liposome dye release assay showing endpoint values compounds with activity against wild-type p7 at 250 nM that were subsequently selected for cell culture studies. Results are the average of two separate experiments with each condition in duplicate and are expressed as a percentage of the untreated protein value following baseline subtraction (liposomes alone). S, solvent control. p7 treated with one rimantadine IC_50_ (40 μM) is included as comparison, novel compounds tested at 250 nM. (B) Inhibition of infectious virus release from Huh7 cells transfected with J4/JFH-1 RNA using novel compounds (400 nM). Selected compounds show minimal effect on intracellular infectivity, replicon replication and have no toxic effects (see Supporting Fig. 5 and Table 2). Chart shows the average released titers from duplicate wells originating from at least two separate experiments, error bars represent standard error between experiments. Rimantadine and the alkylated imino-sugar *N*N-DNJ at ∼IC_50_ are shown for comparison (40 and 20 μM, respectively). (C) Structures of selected initial hit compounds. (D) Predicted docking poses of rimantadine novel compounds at the allosteric binding site (calculated in Glide, Schrodinger). Key interacting residues are highlighted.

The heptameric model was employed as a template for *in silico* compound screening, targeting the principle allosteric site (eHiTS, SymBioSys). 250K compounds were ranked based on predicted binding affinity and drug-like qualities and the top 30 were selected for testing against genotype 1b p7 *in vitro*. Twelve hits showed activity at concentrations >100-fold lower (250 nM) than the rimantadine prototype control (median inhibitory concentration [IC_50_] ∼40 µM) (Fig. 4A). Hits were tested for effects on Huh7 cell culture toxicity and off-target effects against subgenomic luciferase replicons (Fig. S5). 400 nM was selected to provide a minimal 10-fold assay window to test effects on particle secretion.

Selected compounds included adamantanes (LDS3 and 4), yet primarily comprised diverse chemotypes (Fig. 4B,C; Table S2). Effects on HCV particle production were tested against GT1b/2a HCV (J4/JFH-1) 72 hours post-electroporation (Fig. 4B). Aside from LDS17, compounds achieved at least an IC_50_ at 400 nM, 100-fold lower concentration than rimantadine, with many showing more pronounced effects (Fig. 4B). Notably, LDS19 and 21 were markedly more potent, achieving a ∼90-95% reduction in infectious titer.

Exemplars of predicted compound binding site interactions compared with rimantadine are shown (Fig. 4C). Each is distinct, yet overlapping, with the majority of compounds forming H-bond interactions with the backbone carbonyl of Gly46 (e.g., LDS3, 25), the hydroxyl group of Tyr45 (e.g., LDS19, 21, Fig. 6B) or both (e.g., LDS20). LDS24 extends beyond the primary allosteric site into the secondary cavity, where the tetrahydroquinoline portion is predicted to bind to the channel through a pi stacking interaction with Trp30, whereas LDS23 is predicted to bind to the protein through a number of nonpolar Van der Waals contacts. Thus, the p7 allosteric site can be targeted by structurally diverse molecules with variable binding modes, providing considerable scope for future drug discovery.

### Specificity of Potent p7 Targeted Compounds

**Figure 5:**
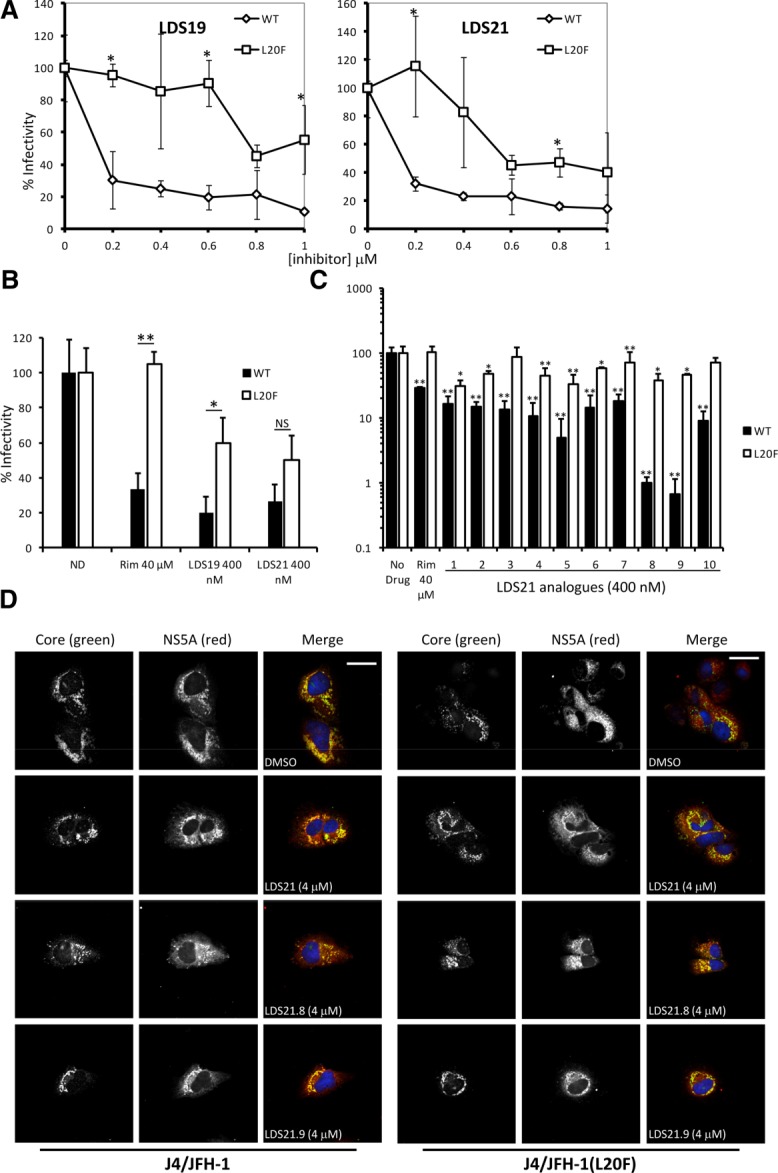
Specificity of novel p7 inhibitors. Screening lead hits LDS19 and 21 were examined for specificity against wild-type and rimantadine resistant HCV. (A) Titration of LDS 19 and 21 against Huh7 cells transfected with both wild-type (diamonds) and Leu20Phe (squares) J4/JFH-1 RNA, showing effects on released infectivity. Graph plots the average released titers from duplicate wells from at least two separate experiments, normalized to the 100% untreated value. Error bars represent normalized percent error of the mean between experiments (*P < 0.05). (B) Effects of p7 inhibitors on HCV spread following infection at low M.O.I (0.0001 FFU/cell). Huh7 cells were infected in triplicate overnight with wild-type (black) and Leu20Phe (white) J4/JFH-1 and then media replaced after extensive washing with that containing inhibitors. Fluorescent NS5A foci were counted at 72 hours postinfection. Histogram shows the average counts for two experiments with triplicate wells normalized to 100% untreated value to allow comparison between viruses. Error bars represent normalized percent error of the mean (*P < 0.05, **P < 0.01). (C) The northern and southern R-groups of LDS21 were rationally altered to determine tolerances for molecular structures and fit within the structure-guided channel model peripheral binding site. Activities of a limited series of LDS21 structural analogs against both wild-type (black) and Leu20Phe (white) J4/JFH-1, normalized to untreated values. Results are the average of two separate experiments with duplicate wells and error bars are normalized percent error of the mean between experiments. (D) Immunofluorescence staining for core (green Alexa-fluor 488 nm) and NS5A (red, Alexa-fluor 565 nm) in huh7 cells 72 hours posttransfection of J4/JFH-1 RNA following treatment with 4 µM LDS21, 21.8, and 21.9.

With hits targeting the primary allosteric site, the rimantadine resistance polymorphism Leu20Phe was predicted to influence binding and so confirm specificity.^15^ LDS19 and 21 were titrated against Huh7 cells transfected with both wildtype and Leu20Phe J4/JFH-1 RNA, and a corresponding right-shift in dose-response curves was evident for the resistant mutant (Fig. 5A). However, submicromolar concentrations of both compounds effectively suppressed Leu20Phe infectious virion production (Fig. 5A), unlike rimantadine, which is ineffective at subcytotoxic concentrations.^15^ Suppression was also apparent during low multiplicity infection, where spread of Leu20Phe HCV was reduced by ∼50-60% in the presence of 400 nM LDS19/21, yet remained insensitive to rimantadine (Fig. 5B). LDS19 and 21 represented attractive starting points for drug development, provided that a robust structure-activity relationship (SAR) could be established. Preliminary modification of LDS21 R-groups was undertaken to assess improvements in potency with reference to predicted interactions with the p7 structure/channel model (Fig. 5C). Ten derivatives cleared MTT and replicon screens and displayed statistically significant improved antiviral effects at 400 nM, with LDS21.8 and 21.9 causing a >2 log_10_ reduction in secreted infectivity (Fig. 5C). Importantly, neither initial hit compounds (data not shown and Fig. S4c) nor LDS21 derivatives caused any obvious defects in HCV protein localization (Fig. 5D; Fig. S4d), polyprotein processing (Fig. S4b), or the accumulation of intracellular infectivity (Fig. S4a), consistent with the specific targeting of the release of infectivity, as mediated by p7 channel activity, confirming studies with prototype inhibitors.^15^

**Figure 6:**
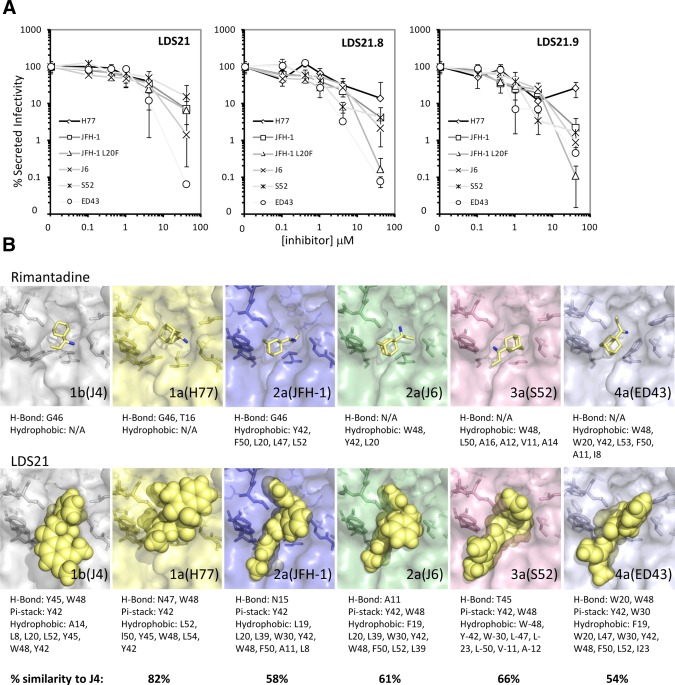
Effects of novel inhibitors against multiple HCV genotypes. (A) LDS21, 21.8, and 21.9 were titrated against Huh7 cells transfected with chimeric HCV RNAs and secreted titers determined by focus forming assay. Results are representative of two independent experiments and are the average of triplicate wells. Error bars represent standard deviations. (B) Channel models for each individual HCV genotype analyzed in (A) were generated in order to predict the influence of polymorphisms upon the binding of rimantadine and LDS21. Changes in sequence strongly influenced the docking poses adopted by molecules, but no clear correlation with inhibitor potency was evident.

### Activities of Genotype 1b-Targeted Compounds Against p7 From Other Genotypes

LDS21, LDS21.8, and LDS21.9 were tested against Huh7 cells transfected with a panel of chimeric HCV RNAs to determine their potential development against other genotypes (Fig. 6A). In general, while IC_50_ values were increased compared with genotype 1b, they remained in the low micromolar range, generating favorable therapeutic ratios in the majority of cases (Table 2). Interestingly, the Leu20Phe polymorphism appeared to confer limited resistance to some novel compounds in the case of genotype 1b, but this was not the case in a genotype 2a JFH-1 context, where it enhanced susceptibility. In addition, genotype 1a appeared more resistant to novel compounds compared with all others tested, despite reasonably high (82%) amino acid conservation with the J4 protein. Interestingly, while IC_50_ and IC_90_ values were close together for genotype 1b, the latter were often much higher in other genotypes, suggesting that effective saturation of the system was harder to achieve in these contexts.

**Table 2:** Therapeutic Indices of Lead LDS Compounds Calculated From Experimental IC50/90 and CC50/90 Values

Compound	Virus	IC50 (μM)	IC90 (μM)	CC50 (μM)	CC90 (μM)	TI(50)	TI(90)
LDS21	J4/JFH-1	0.14	1.09	65.31	199.43	476.72	182.96
	J4/JFH-1(L20F)	0.75	2.18			87.08	91.48
	H77/JFH-1	2.01	62.54			32.49	3.19
	JFH-1	5.4	33.71			12.09	5.92
	JFH-1(L20F)	1.098	27.92			59.48	7.14
	J6/JFH-1	2.14	19.08			30.52	10.45
	S52/JFH-1	3.79	113.77			17.23	1.75
	ED43/JFH-1	2.23	11.27			29.29	17.69
LDS21.8	J4/JFH-1	0.29	0.43	101.36	280.19	349.52	651.6
	J4/JFH-1(L20F)	1.22	1.89			83.08	148.25
	H77/JFH-1	7.41	46.66			13.68	6.00
	JFH-1	1.07	25.83			94.73	10.85
	JFH-1(L20F)	1.95	12.68			51.98	22.097
	J6/JFH-1	0.596	20.04			170.07	13.98
	S52/JFH-1	0.71	24.73			142.76	11.33
	ED43/JFH-1	0.21	9.79			482.67	28.62
LDS21.9	J4/JFH-1	0.34	0.51	406.44	1211.16	1195.41	2374.82
	J4/JFH-1(L20F)	0.45	0.71			903.2	1705.86
	H77/JFH-1	3.03	86.39			134.14	14.02
	JFH-1	0.82	19.82			495.66	61.11
	JFH-1(L20F)	0.44	10.83			923.73	111.83
	J6/JFH-1	1.79	16.83			227.06	71.96
	S52/JFH-1	2.23	16.48			182.26	73.49
	ED43/JFH-1	2.38	11.73			170.77	103.25

To investigate the possible mechanisms underpinning inhibitor effects, we constructed additional channel models for each genotype based on the J4 structure model and assessed the predicted binding of both rimantadine and LDS21 (Fig. 6B). While it was not possible to determine an obvious cause of varied inhibitor effects, it was clear that even minor variations within the peripheral binding site altered the way in which both compounds interacted with the protein. Complete understanding of the basis of genotype-dependent inhibitor sensitivity will likely require more extensive analysis using a wide array of compounds to establish a structure-activity relationship in each instance.

## Discussion

Here we solved a monomeric HCV p7 solution structure, yielding a relevant drug-binding hairpin conformation. In so doing, we validated the presence of an allosteric drug binding cavity reminiscent of that predicted by *in silico* modeling^15^ and demonstrated that this could be exploited for rational development of novel p7 inhibitors. In turn, resultant compound potency not only supports the accuracy and relevance of the structure, but also highlights p7 as a viable target for the development of direct-acting antivirals (DAA).

Our solution structure benefited from the favorable environment provided by MeOH as a solvent which, unlike conditions in previous studies,^24^ allowed a hairpin to form. FLAG-p7 was monodisperse in MeOH, but not in the membrane-mimetic detergent DHPC. This differs from ssNMR studies where DHPC preserved p7 monomers,^25^,^26^ possibly relating to different purification methods^19^,^36^ or even the presence of the FLAG tag. Nevertheless, despite well-dispersed and fully assignable spectra, traditional structure calculation methods could not unambiguously resolve the final p7 hairpin, requiring a novel combination of chemical shift and NOE protocols to reach a satisfactory structural solution. This approach may be of value to further studies of membrane proteins, particularly those containing more than one *trans*-membrane helix. This method is supported by the recent ssNMR structure of the GPCR CXCR1, which was derived using a similar protocol.^37^

The monomer structure showed good agreement with structural/functional studies, including predicted lumen formation by the p7 N-terminal helix,^15^,^23^,^24^,^27^ with alignment of His17, Ser21, and Phe25. Furthermore, the content and orientation of p7 helical domains were reminiscent of those proposed by membrane ssNMR,^26^ suggesting that MeOH induced a protein fold closely resembling that formed in the bilayer. Furthermore, drug interactions in solution and a heptameric structure-model supported a peripheral adamantane binding site analogous to that predicted by *de novo* modeling. Drug binding at this site would likely stabilize closed p7 channels by way of an allosteric mechanism, similar to that proposed in controversial studies of IAV M2.^38^,^39^ However, as rimantadine and other prototype inhibitors are only effective against p7 in the mid-micromolar range,^13^,^15^,^16^ compounds with improved potency tailored to the allosteric site would further support its relevance for antiviral development.

Two additional p7 NMR structures were published during the revision of this article. The first described the structure of a complete genotype 5a hexameric p7 channel complex in DHPC micelles,^40^ with similarity to EM reconstructions of hexameric genotype 2a channels in the same environment.^20^ The unusual structure of this complex could not have been predicted by standard modeling as monomeric hairpins are not present, protomers instead forming an extended “staple-like” structure that interacts with adjacent n+1, 2, and 3 protomers. Accordingly, interhelix distance restraints for individual protomers differ markedly from both our own NOE-defined values, as well as those derived from published ssNMR studies. Furthermore, residues at position 17 and 25 are not oriented toward the lumen, which is significantly wider than models herein, and the polybasic C-terminus of the protein is embedded within the hydrophobic environment. However, in agreement with our studies, a peripheral adamantane site was supported by both NMR and biophysical techniques, although channel formation and drug sensitivity of the 5a channel was not demonstrated.

Given that the monomeric fold of p7 has been extensively characterized as a hairpin, it remains unclear as to how protein rearrangements might occur between the monomer and hexamer forms of the protein. Another possibility is that hexameric and heptameric p7 complexes may involve different p7 monomeric conformations, which could be especially relevant when considering genotype-dependent inhibitor sensitivity individual HCV genotypes appear to favor predominantly one or the other form.^19^,^20^ Unfortunately, we did not include genotype 5a in our genotype analysis, yet its 53% similarity to genotype 1b channels is comparable to that of the 4a protein. It will be of significant interest to establish whether the genotype 5a hexamer structure can similarly be used as a template for structure-guided inhibitor design.

The second reported p7 sNMR structure from the Opella laboratory describes monomeric genotype 1b protein (J4 isolate) within DHPC micelles at pH 4.0 (Cook et al., Biochemistry, 2013, Epub ahead of print). Monomeric stoichiometry was inferred by NMR data analysis, yet why this differs from both our own studies and the Chou study is unclear. Interestingly, this structure also required a combined chemical shift/NOE based protocol as described here. While pdb and NMR data are currently unavailable, the helical composition of the DHPC monomer appears to differ from both our own neutral pH structure, as well as the groups previous ssNMR studies.^26^ It is tempting to speculate that this solution may represent the fold of the J4 protein in the acidified state. Comparison with our structure may therefore provide insight into the gating mechanisms of genotype 1b p7 channel complexes. This study also investigated inhibitor binding, although the peripheral adamantane site was occluded by detergent micelles.

The successful selection of GT1b p7 inhibitors with potent effects *in vitro* and in culture compared to prototypes was surprising, yet this supported the relevance of both the monomeric structure and the derived heptameric channel model. Moreover, improved compound potency corresponded to a much higher degree of predicted fit within the allosteric site compared with rimantadine. Consistent with modeling studies,^15^,^34^ Leu20 resided within the allosteric site on the structure-guided heptamer, allowing the Leu20Phe polymorphism to illustrate site specificity for novel compounds. LDS19 and 21 effectively suppressed Leu20Phe particle release at submicromolar doses, indicative of their improved binding capacities. LDS21 derivatives also displayed activity against other HCV genotypes, yet were less effective against GT1a p7, which we recently showed to display more pore-like behavior compared with 1b channels, which are more M2-like.^28^,^41^ This pore-like shift in equilibrium towards the open state may reduce the number of closed channel complexes to which allosteric inhibitors can bind, although our genotype modeling indicated that varied binding pocket conformations may also influence compound efficacy. This may be further complicated by the formation of either hexameric or heptameric complexes. Nevertheless, LDS21 derivatives demonstrate the potential for variable potency within the series and confirms that molecules tailored to the allosteric site show great potential for drug discovery. Interestingly, the effects of LDS compounds *in vitro* did not necessarily correlate with their effects in culture (Fig. 4). As compounds are administered as a single dose at the beginning of a 72-hour experiment, we predict that differences in cellular uptake and/or turnover may influence the effectiveness of the compounds. Determining metabolic stability will form a critical element of expanding the series.

Skepticism concerning p7 inhibitors arose following clinical trials employing the weak prototype inhibitor, amantadine,^13^,^14^ alongside interferon and ribavirin, with little measurable response.^17^,^18^ This was perhaps unsurprising as interferon-insensitive HCV could continue to replicate and produce escape variants.^42^ Nevertheless, given that even weak selective pressure against p7 can drive HCV to escape, evidenced by the selection of both Leu20Phe^34^ and potentially other polymorphisms,^35^ the prospect of employing targeted, potent molecules becomes highly attractive.

Targeted p7 inhibitors could comprise effective components of interferon-free DAA regimens and suppression of Leu20Phe resistance by LDS19 and 21 suggests that the genetic barrier to HCV escape may be much higher. Interestingly, an amiloride-based genotype 1a p7 inhibitor selected from a bacterial screen, BIT225,^43^ is currently in clinical trials combined with interferon and ribavirin. Early results appear encouraging, albeit in a small number of patients. However, the mode of action for BIT225 is unknown and no published data exist demonstrating its efficacy against HCV in culture. Nevertheless, compounds described herein represent a step-change in potency appropriate for drug discovery. Given recent phase 3 setbacks for some HCV DAAs, it is clear that continued identification and development of drug targets will be critical for the long-term management of HCV disease. Thus, our p7 structure not only represents a valuable tool for investigating channel function, but confirms that p7-targeted drug discovery should provide a further valuable component to future HCV antiviral therapy.

## Abbreviations

DAA: direct-acting antivirals
DHPC: 1,2-diheptanoyl-sn-glycero-3-phosphocholine
GAPDH: glyceraldehyde 3-phosphate dehydrogenase
GT: genotype
HCC: hepatocellular carcinoma
HCV: hepatitis C virus
IAV: influenza A virus
M2: matrix protein 2
MD: molecular dynamics
MeOH: methanol
MTT: 3-(4,5-Dimethylthiazol-2-yl)−2,5-diphenyltetrazolium bromide
native PAGE: native polyacrylamide gel electrophoresis
NOE: nuclear Overhauser effect
NS: nonstructural
PRE: paramagnetic relaxation enhancement
rp-HPLC: reverse phase high performance liquid chromatography
SAR: structure-activity relationship
sNMR: solution nuclear magnetic resonance
ssNMR: solid-state NMR
svAUC: sedimentation velocity analytical ultracentrifugation
TFE: tri-fluoro-ethanol
TM: trans-membrane

## Supporting Information

Supporting Figure 1: Characterisation of isotopically labelled, monomeric p7 protein for solution NMR studies. Isotopically labelled FLAG-tagged p7 from the J4 isolate (genotype 1b) was expressed in *E. coli* as a glutathione-S-transferase fusion, followed by cleavage and purification by reverse phase HPLC. **A.** Functionality of isotopically labelled FLAG-p7 was demonstrated using liposome dye release assays in the presence of increasing concentrations of the adamantane p7 inhibitor, rimantadine. Solid black line shows dye release in the absence of drug, dashed lines represent increasing drug concentrations from darkest to lightest (10, 20, 40 and 80 µM). L, liposomes only; S, solvent control (5 µl methanol and 1 µl DMSO). **B.** Intrinsic tryptophan fluorescence in the presence/absence of acrylamide quencher was determined for p7 reconstituted in either methanol, LMPG detergent, DHPC detergent or liposomes. Results are shown as a Stern-Volmer plot whereby the gradient of the line is indicative of the difference between quenched and unquenched states. Low values for liposomes and DHPC indicate that residues in p7 were occluded as part of an oligomeric complex, whereas high values in methanol indicate that in the absence of quencher residues were exposed with resultant increase in fluorescence. LMPG induces partial oligomerisation and so gives an intermediate value. **C.** Methanol-reconstituted FLAG-p7 was subjected to sedimentation velocity analytical ultracentrifugation and was shown to migrate as a monodisperse population with estimated mass of 9.71 kDa. **D.** p7 reconstituted in methanol or DHPC was subjected to CD spectroscopy to determine alpha helical content, with both giving a similar pattern of folding. Methanol-solubilised p7 was monomeric by SDS-PAGE, however, low concentrations of DHPC induced the formation of oligomeric species, consistent with B above. **E.** PRE measurements of MTSL “spin-labelled” p7 were determined in the oxidised or reduced states, with resultant ratios representing inter-molecular distances plotted as a histogram. Internally labelled protein (Cys 27) produced an asymmetrical pattern of interaction (ratio value <1) consistent with the residue being present in proximity to the inter-helical basic loop, whereas N-terminally labelled protein (Cys −1) displayed interactions with both adjacent residues and the C-terminal region of the protein, indicative of the formation of a hairpin structure. The structure of MTSL and the modified Cys residues are shown.

Supporting Figure 2: NMR spectral data. Isotopically labelled p7 produced well dispersed spectra consistent with an alpha-helical fold. **A.** Ensemble of twelve lowest energy structures calculated in ARIA 2.3 solely based on NOE and TALOS restraints starting from an extended structure. **B.** Refined ensemble of twenty structures from cs-memrosetta refined with NOE’s using Aria 2.3. **C.** Diagrammatic representation of long distance NOE restraints in lowest energy structure. **B.** Fully assigned ^1^H/^15^N HSQC spectrum of p7 in its monomeric state in methanol. **C.** Strip plot showing inter-residue connectivities.

Supporting Figure 3: Quantification of shift metric (Δppm) for p7 residues upon binding to rimantadine in solution (see methods). Shifts >0.14 were taken to be significant and are indicated in red.

Supporting Figure 4: Off-target effects in Huh7 cells for novel p7 inhibitors. All compounds failing screening criteria were excluded from virion secretion assays. **A.** Intracellular infectivity within JFH-1 transfected Huh7 cells harvested by freeze-thaw, 72 hr post transfection, in presence of increasing concentrations of LDS21, LDS21.8 and LDS21.9. Results are representative of two separate experiments with triplicate wells, error bars represent standard deviations. **B.** Polyprotein expression and processing within inhibitor treated cells (400 nM). Whole cell lysates were probed for viral and cellular proteins as indicated by western blot 72 hr post-transfection. NB GT1b p7 specific antibody supplies have been exhausted. **C.** Immunofluorescence staining of core and NS5A proteins within Huh7 cells transfected with JFH-1 RNA at 72 hr post-transfection.

Supporting Figure 5: Effects of novel inhibitors on Huh7 cell viability (MTT assay) and HCV RNA replication (JFH-1 luciferase subgenomic replicon). Huh7s/replicon cells were incubated with increasing concentrations of inhibitors for 72 hr or DMSO as a control prior to processing for MTT/luciferase assays (see methods). Results are the average of triplicate wells and are representative of three independent experiments. Error bars represent standard deviations.

Supporting Table: Compound structures for hits from initial screen
