# Supplementary figures and images for: Structure-Guided Design Affirms Inhibitors of Hepatitis C Virus p7 as a Viable Class of Antivirals Targeting Virion Release
Source: Hepatology. 2013 Dec 24;59(2):408–22. doi: 10.1002/hep.26685 (PMC4298801; doi:10.1002/hep.26685)

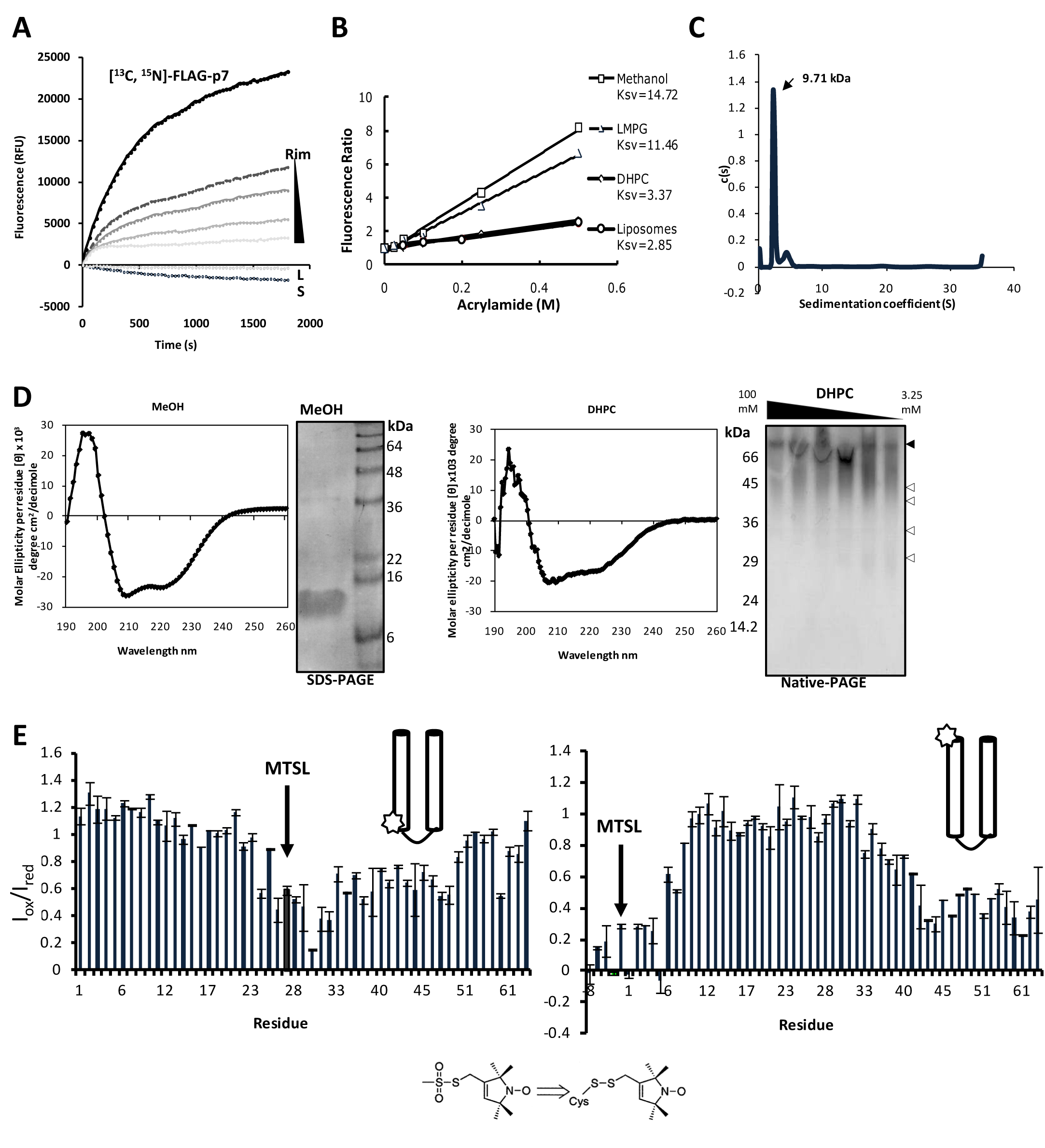

Supplement: Supplementary file 1 — Supporting Figure 1: Characterisation of isotopically labelled, monomeric p7 protein for solution NMR studies. Isotopically labelled FLAG-tagged p7 from the J4 isolate (genotype 1b) was expressed in E. coli as a glutathione-S-transferase fusion, followed by cleavage and purification by reverse phase HPLC. A. Functionality of isotopically labelled FLAG-p7 was demonstrated using liposome dye release assays in the presence of increasing concentrations of the adamantane p7 inhibitor, rimantadine. Solid black line shows dye release in the absence of drug, dashed lines represent increasing drug concentrations from darkest to lightest (10, 20, 40 and 80 µM). L, liposomes only; S, solvent control (5 µl methanol and 1 µl DMSO). B. Intrinsic tryptophan fluorescence in the presence/absence of acrylamide quencher was determined for p7 reconstituted in either methanol, LMPG detergent, DHPC detergent or liposomes. Results are shown as a Stern-Volmer plot whereby the gradient of the line is indicative of the difference between quenched and unquenched states. Low values for liposomes and DHPC indicate that residues in p7 were occluded as part of an oligomeric complex, whereas high values in methanol indicate that in the absence of quencher residues were exposed with resultant increase in fluorescence. LMPG induces partial oligomerisation and so gives an intermediate value. C. Methanol-reconstituted FLAG-p7 was subjected to sedimentation velocity analytical ultracentrifugation and was shown to migrate as a monodisperse population with estimated mass of 9.71 kDa. D. p7 reconstituted in methanol or DHPC was subjected to CD spectroscopy to determine alpha helical content, with both giving a similar pattern of folding. Methanol-solubilised p7 was monomeric by SDS-PAGE, however, low concentrations of DHPC induced the formation of oligomeric species, consistent with B above. E. PRE measurements of MTSL “spin-labelled” p7 were determined in the oxidised or reduced states, with resultant ra [file hep0059-0408-sd1.tif]

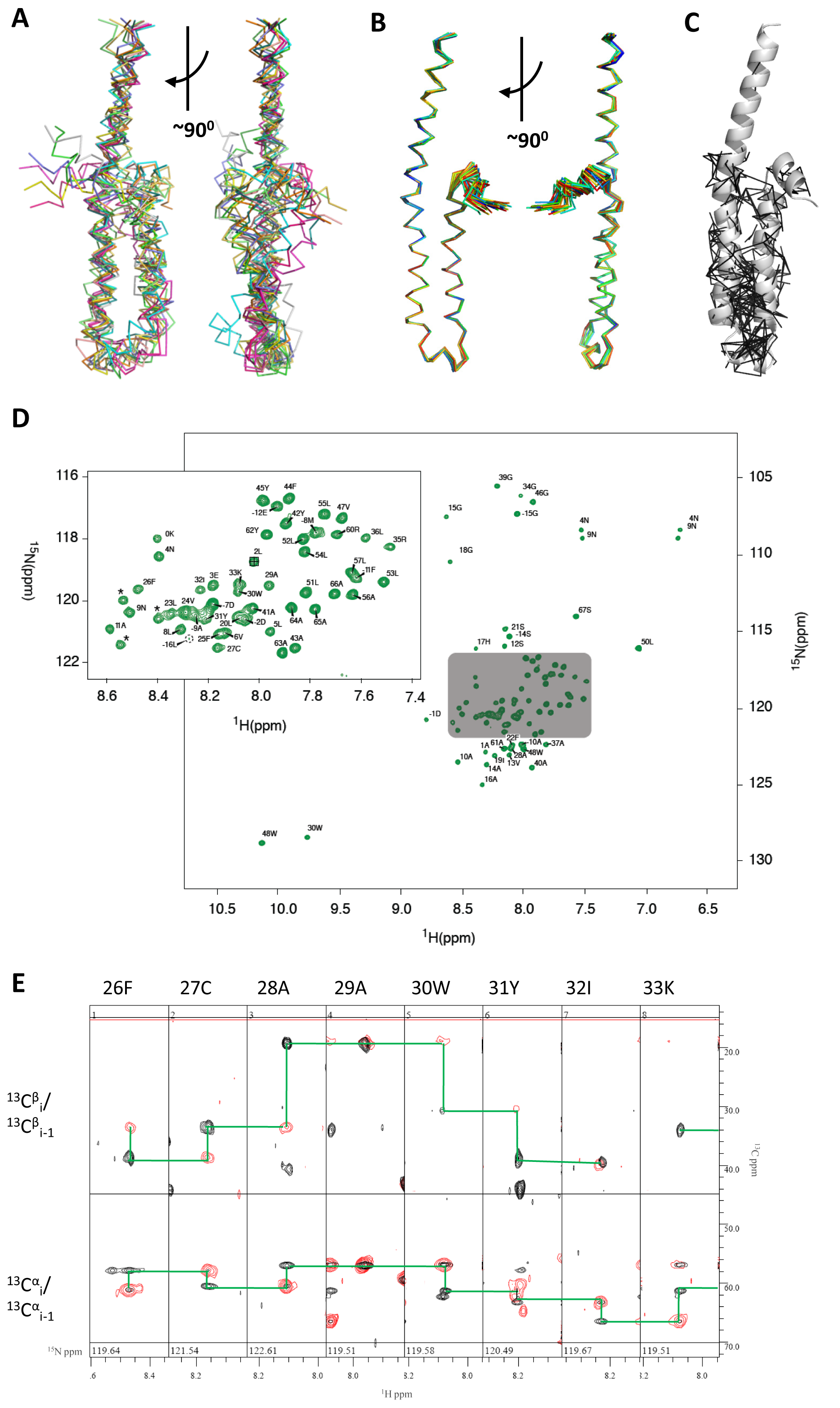

Supplement: Supplementary file 2 — Supporting Figure 2: NMR spectral data. Isotopically labelled p7 produced well dispersed spectra consistent with an alpha-helical fold. A. Ensemble of twelve lowest energy structures calculated in ARIA 2.3 solely based on NOE and TALOS restraints starting from an extended structure. B. Refined ensemble of twenty structures from cs-memrosetta refined with NOE’s using Aria 2.3. C. Diagrammatic representation of long distance NOE restraints in lowest energy structure. B. Fully assigned 1H/15N HSQC spectrum of p7 in its monomeric state in methanol. C. Strip plot showing inter-residue connectivities. [file hep0059-0408-sd2.tif]

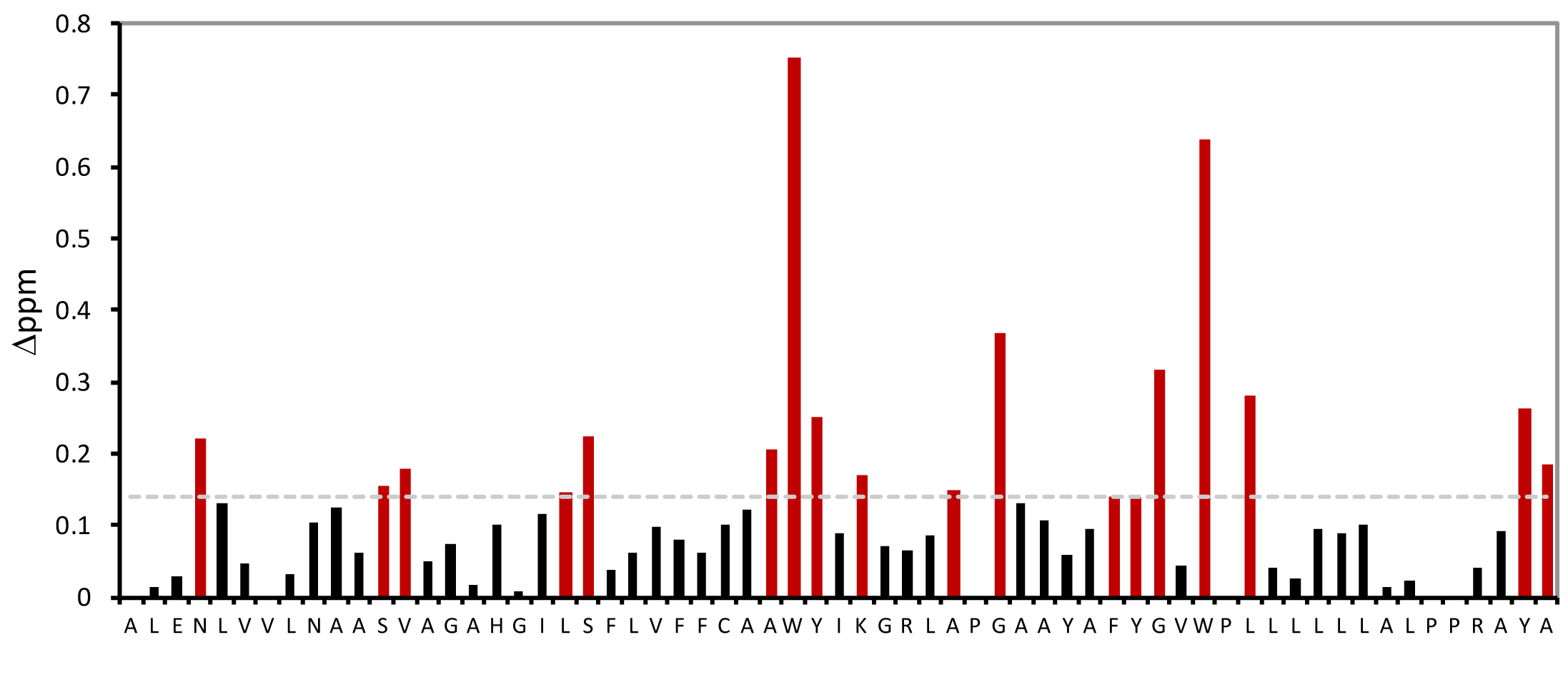

Supplement: Supplementary file 3 — Supporting Figure 3: Quantification of shift metric (Δppm) for p7 residues upon binding to rimantadine in solution (see methods). Shifts >0.14 were taken to be significant and are indicated in red. [file hep0059-0408-sd3.tif]

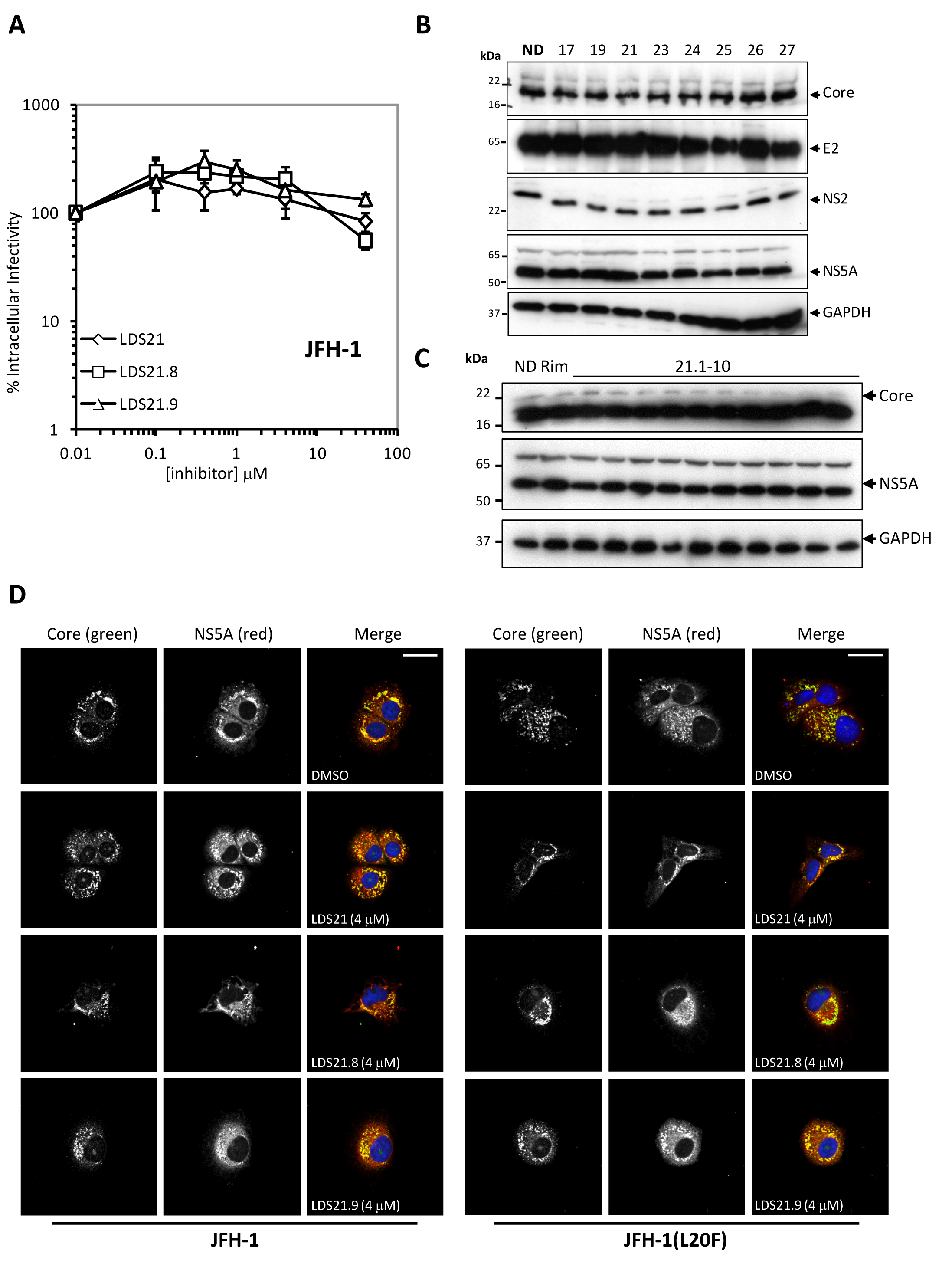

Supplement: Supplementary file 4 — Supporting Figure 4: Off-target effects in Huh7 cells for novel p7 inhibitors. All compounds failing screening criteria were excluded from virion secretion assays. A. Intracellular infectivity within JFH-1 transfected Huh7 cells harvested by freeze-thaw, 72 hr post transfection, in presence of increasing concentrations of LDS21, LDS21.8 and LDS21.9. Results are representative of two separate experiments with triplicate wells, error bars represent standard deviations. B. Polyprotein expression and processing within inhibitor treated cells (400 nM). Whole cell lysates were probed for viral and cellular proteins as indicated by western blot 72 hr post-transfection. NB GT1b p7 specific antibody supplies have been exhausted. C. Immunofluorescence staining of core and NS5A proteins within Huh7 cells transfected with JFH-1 RNA at 72 hr post-transfection. [file hep0059-0408-sd4.tif]

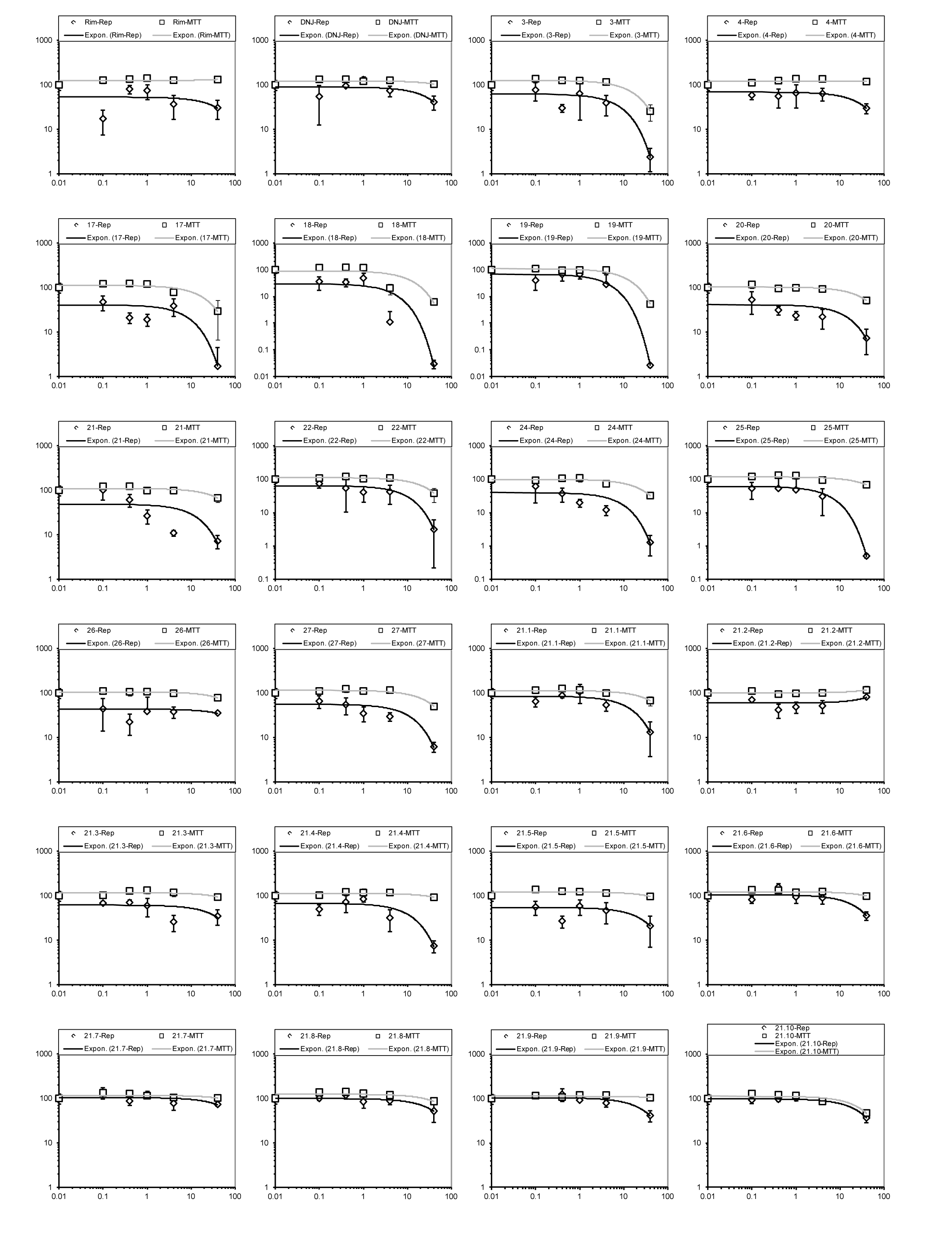

Supplement: Supplementary file 5 — Supporting Figure 5: Effects of novel inhibitors on Huh7 cell viability (MTT assay) and HCV RNA replication (JFH-1 luciferase subgenomic replicon). Huh7s/replicon cells were incubated with increasing concentrations of inhibitors for 72 hr or DMSO as a control prior to processing for MTT/luciferase assays (see methods). Results are the average of triplicate wells and are representative of three independent experiments. Error bars represent standard deviations. [file hep0059-0408-sd5.tif]
