# Supplementary material for: Structure-Guided Design Affirms Inhibitors of Hepatitis C Virus p7 as a Viable Class of Antivirals Targeting Virion Release
Source: Hepatology. 2013 Dec 24;59(2):408–22. doi: 10.1002/hep.26685 (PMC4298801; doi:10.1002/hep.26685)
Supplement: Supplementary file 6 — Supporting Table: Compound structures for hits from initial screen [file hep0059-0408-sd6.docx]

**Supplementary Table: Compound structures for hits from initial screen**

| *Compound* | *Structure* |
| --- | --- |
| LDS3 |  |
| LDS4 |  |
| LDS17 |  |
| LDS18 |  |
| LDS19 | Not shown |
| LDS20 |  |
| LDS21 | Not shown |
| LDS23 |  |
| LDS24 |  |
| LDS25 |    |
| LDS26 |  |
| LDS27 |  |
