# Supplementary material for: Structure-Guided Design Affirms Inhibitors of Hepatitis C Virus p7 as a Viable Class of Antivirals Targeting Virion Release
Source: Hepatology. 2013 Dec 24;59(2):408–22. doi: 10.1002/hep.26685 (PMC4298801; doi:10.1002/hep.26685)
Supplement: Supplementary file 7 — Supporting Information [file hep0059-0408-sd7.docx]

**Supplementary** **Experimental Procedures**

**NMR spectroscopy.** Spectra were recorded using Varian Inova 500, 600 or 750 (cold-probe) MHz (Varian Inc., CA) spectrometers at 25 °C. Lyophilised [^13^C, ^15^N]-FLAG-p7 samples were reconstituted in 100 % MeOH at 0.3-0.6 mM. Backbone assignment employed 3D triple resonance experiments HNCA, HN(CO)CA, HN(CA)CB, CBCA(CO)NH and HNCO. Side-chain chemical shifts were derived from 3D C(CO)NH, H(CCO)NH, H(C)CH-COSY, H(C)CH-TOCSY, CCH-TOCSY, 2D ^1^H-^13^C- HSQC experiments and 2D 70ms ^1^H-^1^H Watergate TOCSY. Aromatic group assignments were obtained from 2D CB(CGCD)HD, CB(CGCDCE)HE and intra-residual NOE’s in a 2D 120 ms Watergate NOESY. NOEs were obtained from 3D ^1^H -^15^N NOESY-HSQC, ^1^H -^13^C NOESY-HSQC and 2D ^1^H-^1^H Watergate NOESY experiments, mixing time 120 ms. Data were processed using NMRPipe. A cosine bell function, followed by zero filling and Fourier transformation was used. 3D experiments used linear prediction in the ^15^N dimension to double the number of points. Spectra were plotted in CCPNmr Analysis (W.Boucher, T.Stevens). ^1^H-^15^N-HSQC experiments +/-10 mM rimantadine were performed at 25 °C as above. Chemical shift metrics were calculated from apo/holo ^1^H-^15^N-HSQC: Δ=[(δ^15^N_apo_/_holo_)2+(5×δ^1^H_apo_/_holo_)2]0.5^(^[^1^](#_ENREF_1)^,^ [^2^](#_ENREF_2)^)^.

**Calculation of NMR structures with cs-memrosetta.** Structures were calculated using a membrane chemical shift protocol ‘cs-memrosetta’ which uses the memrosetta^(^[^3-5^](#_ENREF_3)^)^ (membrane rosetta version 3.1) force field for Monte-Carlo structure calculation and refinement in place of the standard rosetta 3 force field^(^[^6^](#_ENREF_6)^)^. The cs-memrosetta protocol uses the same 3 stages approach used for cs-rosetta^(^[^7^](#_ENREF_7)^)^ calculations: fragment selection, structure calculation, scoring. However, in the fragment selection stage 9 and 3 residue fragments compatible with the observed HN, N, Cα, Cβ and CO chemical shifts were selected using the standard cs-rosetta methodology. During structure calculation *trans*-membrane segments, inner and outer loops, and tails for input into the cs-memrosetta force field were calculated using Octopus^(^[^8^](#_ENREF_8)^)^ and lipophilicity scores were calculated by rosetta. 2000 decoys were calculated (memrosetta). No restraints were placed on helix - membrane angles, *trans*-membrane segment helical content, or embedding of N or C termini in the *trans*-membrane segment, other than those provided by the chemical shift fragment selection and no interpolation between the two layers for the pair-term was used. Final stage decoys were scored against both chemical shifts and force-field energy using the cs-rosetta protocol. Two clusters of low energy structures were observed in the final ensemble. The lowest energy decoy and half the low energy decoys calculated had consistent hairpin structures; 20 were used for further refinement (backbone rmsd for residues 1-40, 55-70 2.08 Å). The remaining structures were more globular, disordered (backbone rmsd vs mean for residues 1-40, 55-70, 3.6 Å), inconsistent with observed unique nOe’s and so rejected.

**Refinement of cs-memrosetta structures using Aria 2.3.** Cs-memrosetta structures were refined against observed nOes with automated peak assignment using a semi-rigid dynamics protocol, implemented using Aria 2.3^(^[^9^](#_ENREF_9)^)^. During simulated annealing the regions of *trans*-membrane helical secondary structure identified by cs-memrosetta and TALOS (residues 5-43, 56-74 & 77-84, tagged p7 sequence^(^[^10^](#_ENREF_10)^)^) were restrained as semi rigid secondary structure elements by a network of distance restraints between backbone N, CA, C' and O atoms, which constrained their relative distances within the elements to be within ±0.3 Å of those found in the lowest energy cs-memrosetta structure. This allowed retention of the backbone conformation of the secondary structure elements, whilst allowing all other degrees of freedom to evolve during the Aria protocol. Semi-rigid restraints were not applied to the loop region 25-36 (p7 sequence). 104 TALOS Ramachandran dihedral angle restraints (52 phi and 52 psi restraints) were used throughout. nOe restraints were added over 3 refinement rounds in a hierarchical protocol, ordered by dispersion and nOe data quality: aromatic, amide backbone, then aliphatic side-chains. In each calculation round, previous restraints were frozen and automated assignments were only made on the newly added restraints. During each round of Aria refinement the protocol was seeded with the lowest energy membrane cs-memrosetta structure as a template to allow the creation of an initial nOe list compatible with the cs-memrosetta structures and the cs-memrosetta ensemble was added to the calculation as iteration 0 (it0) with no floating stereo-specific assignments. The weight or assignment parameter was modified to 0.986, 0.983 and 0.982 for rounds 1-3^(^[^11^](#_ENREF_11)^)^. Extended slow cooling periods were used throughout, as described^(^[^12^](#_ENREF_12)^)^, to aid convergence. Explicit water refinement was not carried out at the end of the protocol. Calculation without the semi-rigid restraints using the lowest energy cs-memrosetta structure as a template for nOe assignment, consistently resulted in hair pin structures, however, the orientation and regularity of the secondary structure was lost due to poor chemical shift dispersion available for nOe assignments (figure 1a, b). Ramachandran statistics (Molprobity^(^[^13^](#_ENREF_13)^)^): 94.5% (1568/1660) residues in favoured (98%) regions, 98.7% (1639/1660) residues in allowed (>99.8%) regions, average of 1.05 outliers per model (52 GLY (18) 45 CYS (3)).

**Circular Dichroism (CD) of FLAG-p7***.* Far UV CD spectroscopy was conducted on 0.25-0.3 mg/ml FLAG-p7 in 100 % methanol or 300 mM DHPC (1,2-diheptanoyl-*sn*-glycero-3-phosphocholine), 20 mM sodium phosphate [pH 7.0]. Spectra were collected at 1 nm intervals using a JASCO J175 spectropolarimeter with a Peltier thermostat, in a 1 mm path-length quartz cuvette over the wavelength range 190-260 nm. Baseline-corrected measurements were averaged over 3 scans at 25 °C, with constant nitrogen flushing.

**Tryptophan fluorescence spectroscopy**. Spectra (50 µM FLAG-p7) were recorded on a Fluorolog®-3 (HORIBA JOBIN YVON) spectrometer (25 °C, 3 nm monochromator slit widths, 10 mm cell, 295 nm light) for [^13^C, ^15^N]-FLAG-p7 in 100 % methanol, 300 mM detergent (DHPC or 1-myristoyl-2-hydroxy-*sn*-glycero-3-[phospho-RAC-(1-glycerol)], LMPG) or 50 μM liposomes (1:1 DOPA/DOPC). Emission spectra were recorded between 290 nm and 500 nm. Aliquots of 5 M acrylamide (Sigma Aldrich) in 6 M Guanidinium chloride, 20 mM sodium phosphate [pH 7.0] were added and sequential fluorescence spectra were recorded. Quenching was described by the Stern-Volmer relationship (*F_0_/F* = *1 + K_SV_*[*Q*]; *F* and *F_0_* = unquenched/quenched intensities; *K_SV_* = Stern-Volmer constant, *Q* = quencher concentration). Linear Stern-Volmer plots indicate quenching of a homogenous fluorophore population.

**Sedimentation Velocity Analytical Ultracentrifugation (svAUC).** svAUC of 0.1 mM [^13^C, ^15^N]-FLAG-p7 was conducted at 20.0 °C, referenced to 100 % methanol. 400 µl samples were centrifuged in 1.2 cm pathlength 2-sector aluminium centrepiece cells with sapphire windows in a 4-place An60 Ti analytical rotor, Optima XL-I analytical ultracentrifuge (Beckman) at 40 000 rpm. Changes in solute concentration were detected by absorbance (280nm), a total of 197 scans were collected over approximately 20 hours. Buffer densities and viscosities were calculated using the programme Sednterp version 1.09 (2006, Dr. Thomas Laue, University of New Hampshire). Radial absorbance plots were used for fitting to sedimentation profiles with the program Sedfit, version 12.1b (2010), using a continuous distribution c(s) Lamm equation model.

**FLAG-p7 native PAGE***.* Conducted as described^(^[^14^](#_ENREF_14)^)^.

**Paramagnetic relaxation enhancement (PRE).** Wild type and mutant (Cys0, C27S, details upon request) [^13^C, ^15^N]-FLAG-p7 were solubilised in DMSO and modified with a molar ratio of 3:1 MTSL (*S*-(2,2,5,5-tetramethyl-2,5-dihydro-1H-pyrrol-3-yl)methyl methanesulfonothioate) at room temperature for 12-16 h, yielding a cysteine disulphide-linked nitroxide spin label. Proteins were re-purified (rp-HPLC), lyophilised then solubilised in 100 % MeOH. ^1^H-^15^N-HSQC experiments were acquired at 25°C (600 MHz), then repeated following equilibration in 2-3 fold molar excess of ascorbic acid (<1% sample dilution). Spectra were analysed in nmrPipe and nmrDraw (nlinLS), determining intensity ratios of cross-peaks in oxidized (paramagnetic) versus reduced (diamagnetic) spectra.

**References**

1. Williamson RA, Carr MD, Frenkiel TA, Feeney J, Freedman RB. Mapping the binding site for matrix metalloproteinase on the N-terminal domain of the tissue inhibitor of metalloproteinases-2 by NMR chemical shift perturbation. Biochemistry 1997;36:13882-13889.

2. Kalverda AP, Thompson GS, Vogel A, Schroder M, Bowie AG, Khan AR, Homans SW. Poxvirus K7 protein adopts a Bcl-2 fold: biochemical mapping of its interactions with human DEAD box RNA helicase DDX3. Journal of molecular biology 2009;385:843-853.

3. Barth P, Wallner B, Baker D. Prediction of membrane protein structures with complex topologies using limited constraints. Proceedings of the National Academy of Sciences of the United States of America 2009;106:1409-1414.

4. Barth P, Schonbrun J, Baker D. Toward high-resolution prediction and design of transmembrane helical protein structures. Proceedings of the National Academy of Sciences of the United States of America 2007;104:15682-15687.

5. Yarov-Yarovoy V, Schonbrun J, Baker D. Multipass membrane protein structure prediction using Rosetta. Proteins 2006;62:1010-1025.

6. Bradley P, Misura KM, Baker D. Toward high-resolution de novo structure prediction for small proteins. Science 2005;309:1868-1871.

7. Shen Y, Lange O, Delaglio F, Rossi P, Aramini JM, Liu G, Eletsky A, et al. Consistent blind protein structure generation from NMR chemical shift data. Proceedings of the National Academy of Sciences of the United States of America 2008;105:4685-4690.

8. Viklund H, Elofsson A. OCTOPUS: improving topology prediction by two-track ANN-based preference scores and an extended topological grammar. Bioinformatics 2008;24:1662-1668.

9. Rieping W, Habeck M, Bardiaux B, Bernard A, Malliavin TE, Nilges M. ARIA2: automated NOE assignment and data integration in NMR structure calculation. Bioinformatics 2007;23:381-382.

10. Clarke D, Griffin S, Beales L, Gelais CS, Burgess S, Harris M, Rowlands D. Evidence for the formation of a heptameric ion channel complex by the hepatitis C virus p7 protein in vitro. The Journal of biological chemistry 2006;281:37057-37068.

11. Linge JP, O'Donoghue SI, Nilges M. Automated assignment of ambiguous nuclear overhauser effects with ARIA. Methods in enzymology 2001;339:71-90.

12. Fossi M, Oschkinat H, Nilges M, Ball LJ. Quantitative study of the effects of chemical shift tolerances and rates of SA cooling on structure calculation from automatically assigned NOE data. Journal of magnetic resonance 2005;175:92-102.

13. Chen VB, Arendall WB, 3rd, Headd JJ, Keedy DA, Immormino RM, Kapral GJ, Murray LW, et al. MolProbity: all-atom structure validation for macromolecular crystallography. Acta crystallographica. Section D, Biological crystallography 2010;66:12-21.

14. Foster TL, Verow M, Wozniak AL, Bentham MJ, Thompson J, Atkins E, Weinman SA, et al. Resistance mutations define specific antiviral effects for inhibitors of the hepatitis C virus p7 ion channel. Hepatology 2011;54:79-90.
